# Recovery housing for substance use disorder: a systematic review

**DOI:** 10.3389/fpubh.2025.1506412

**Published:** 2025-03-06

**Authors:** Corrie L. Vilsaint, Alex G. Tansey, Emily A. Hennessy, David Eddie, Lauren A. Hoffman, John F. Kelly

**Affiliations:** ^1^Recovery Research Institute, Center for Addiction Medicine, Massachusetts General Hospital, Boston, MA, United States; ^2^Department of Psychiatry, Harvard Medical School, Boston, MA, United States

**Keywords:** recovery housing, recovery residence, Oxford house, sober home, systematic review, substance use, opioid use, alcohol use

## Abstract

Recovery housing, an abstinence-based living environment, is the most widely available form of substance use disorder (SUD) recovery support infrastructure. This systematic review characterized the randomized control trials (RCT) and quasi-experimental designs (QED) research on recovery housing. We conducted a search across PubMed, EMBASE, CINAHL, PsycINFO and CENTRAL published prior to February 2024. For inclusion, studies had to compare recovery housing alone to a non-recovery housing condition. Our search identified 5 eligible studies including 3 RCTs and 2 QEDs, across 11 reports. Participants Ns ranged from 150 to 470 and follow-up durations were 6–24 months. Recovery housing interventions performed better than continuing care as usual/no intervention on abstinence, income, employment, criminal charges and to a lesser extent incarceration. Recovery housing also performed better than comparative interventions delivered in other types of residential settings (e.g., therapeutic communities) on increasing alcohol abstinence and reducing days of substance use, while also increasing income and employment rates. An exception was in study samples that had high percentages of formerly incarcerated women (90% or more) where reduced substance use was the only benefit of recovery housing when compared to other types of residential interventions and was inconsistent when compared to continuing care as usual/no intervention. Moreover, recovery housing demonstrated higher cost effectiveness than continuing care as usual/no intervention and comparative interventions. Based on quantity, quality, and support for the service, the existing level of evidence for recovery housing is considered moderate. Expanding access to recovery housing may enhance outcomes for individuals with SUD, in general, while producing cost saving benefits, but given the small number of high quality studies additional comparative trials are needed. Also, future research should identify specific sub-groups who may or may not benefit from recovery housing interventions and why, so as to develop and test suitably augmented housing models or identify helpful alternatives.

## 1 Introduction

Substance use disorder (SUD) is characterized by continued substance use despite harmful consequences including clinically significant impairment and/or distress. It remains one of the world’s greatest public health problems, with 48 million people living with SUD in the United States, with more than 1 in 100 experiencing this worldwide (1, 2). In the United States, drugs contribute to around 300 fatal overdose deaths per day (3) and excessive alcohol use contributes to 480 deaths per day (4). In addition to high mortality rates, SUD is associated with a loss of approximately 25 disability-adjusted life years per affected person (4). The economic cost of illicit drug use and excessive alcohol use in the United States is over 400 billion annually (5–8).

Despite the immense global impact of SUD, this condition has a relatively good prognosis, with 70–75% of people seeking SUD recovery eventually achieving sustained SUD remission (9, 10). While some achieve SUD remission in their initial recovery attempt, most require multiple attempts, (5, 9) and it is not until around 5 years of sustained remission that an individual’s risk of a reoccurrence falls to below the 15% population probability of developing SUD in the subsequent year (9, 11, 51).

A rapidly growing literature highlights the importance for longitudinal care and management of SUD, consistent with care models for other chronic health conditions. This literature indicates that SUD relapse risk is significantly ameliorated by long-term recovery support that extends beyond current standard SUD-care practices like medically managed withdrawal and 28-day residential treatment programs. Although many forms of ongoing support may benefit individuals seeking SUD recovery, one of the most important and widely available is recovery housing. Recovery housing has markedly grown in availability and utilization over the past decade and is now the most widely available form of SUD recovery support infrastructure (12), with 10,000 recovery houses across the United States (13).

Recovery housing (also commonly referred to as recovery residences) is an umbrella term referring to abstinence-based living environments that offer short- or long-term instrumental and social support for individuals seeking recovery from SUD (14). Recovery houses are distinct from housing offered within inpatient/residential treatment and offer a range of levels of support that have been organized, described, and certified (15). At one end of the spectrum, in addition to providing a safe living environment, they may offer a high degree of structure and onsite clinical services (level 4/type C). Alternatively, recovery houses can be supervised by a paid house manager, include administrative support, and oversee the peer recovery support staff (level 3/type S), or provide management by an appointed resident house leader (level 2/type M). At the other end, recovery housing can be peer-run residences with little formal structure or supervision (14, 16). Oxford Houses are an example of this latter form of recovery housing. In the Oxford House model, residents share in decision making, house management, and informally support one another by sharing experience-informed advice around health care, employment, management of legal problems, and navigating social service systems (7).

Recently revised care guidelines to recommend recovery housing as part of the SUD care continua (16) suggest the value added by recovery housing is significant. And at the same time, over a decade has passed since the last systematic review of recovery housing (17) which is well beyond the recommended 2 years to ensure up-to-datedness of review findings (18). A more recent systematic quantification of the state of the science on recovery housing can inform policy and help guide clinicians, individuals, and their families with decision making around these services. The purpose of this systematic review is to summarize the existing recovery housing literature on housing residents’ recovery outcomes (e.g., substance use, employment, recovery capital) and housing cost-effectiveness. We also draw attention to current knowledge gaps in this literature that can inform future research.

## 2 Method

### 2.1 Search strategy

We conducted a systematic search of the literature published prior to February 2, 2024, using the search terms related to recovery housing (e.g., ‘communal living’, ‘sober house’, “transitional home”) and substance use terms (e.g., ‘substance use disorder,’ ‘alcohol use disorder,’ ‘drug use disorder,’ ‘cannabis,’ ‘heroin,’ ‘opioids’). See Appendix A for the full syntax.

### 2.2 Inclusion criteria

This review focuses on studies with a comparison group to draw the most rigorous conclusions about recovery housing efficacy. We included peer-reviewed, quantitative studies that longitudinally compared recovery housing alone to a non-recovery housing condition, including randomized controlled trials (RCT) and quasi-experimental designs (QED) of two pre-existing groups. All outcomes related to residents’ recovery outcomes were included along with results from cost-to-benefit analyses. We conceptualized recovery outcomes very broadly to ensure that all potentially relevant outcomes were captured and anticipated finding outcomes related to specific constructs often measured (substance use, employment, service usage) and broader recovery constructs (e.g., life satisfaction, recovery capital). We excluded studies: (1) of recovery housing that were not on the care continua for SUD (e.g., target population was unhoused individuals as opposed to individuals with substance use problems) and (2) where the populations were institutionalized at the time of the study (e.g., criminal legal system). We also did not include outcomes which were the result of mediators and moderator analysis, although the study reporting them was eligible provided they examined the main effect of recovery housing on outcomes. Given resource constraints, as well as the focus on recovery residences within the context of high-income countries to best inform policy and practice in these regions (e.g., North America), studies published in languages other than English were also excluded.

**Figure 1:**
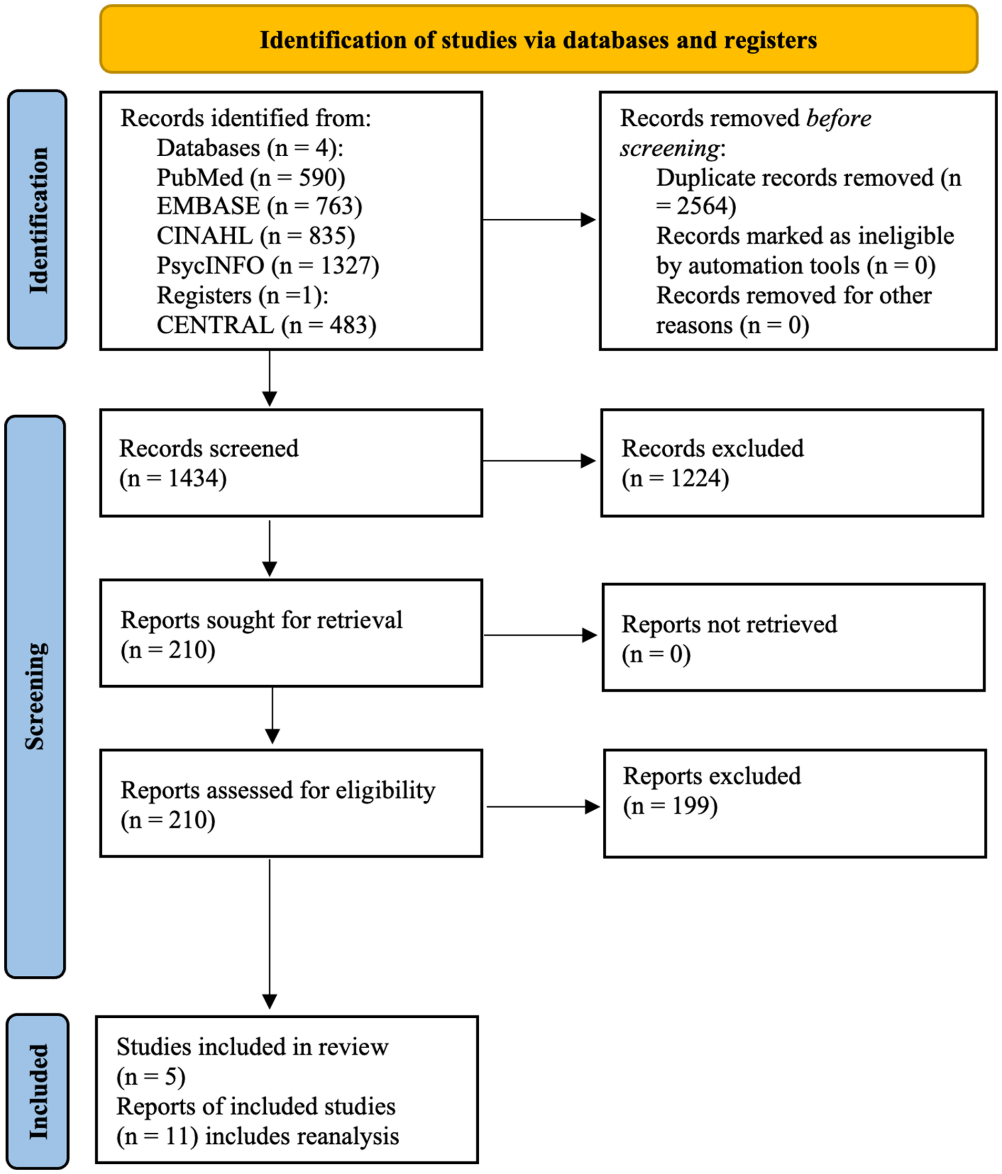
Systematic review of the evidence on recovery housing flow diagram.

Based on these search criteria, we identified 3,998 records across four publicly available databases (i.e., PubMed, EMBASE, CINAHL, and PsycINFO) and one register (CENTRAL) from which Endnote software identified 2,564 duplicate records to be removed. Using a single reviewer (first author, CV), the title screening process excluded 736 records. An abstract review excluded an additional 404 records, and a full-text review excluded another 84 records. This process resulted in 210 full-text reports for further review. The PRISMA study flow diagram is displayed in Figure 1 (19, 20). A single reviewer (first author, CV) extracted all data. These tables were then used to support the descriptive summary. The review included a total of five studies: three RCTs and two QEDs.

## 3 Results

Of the 210 full text reports, 96 described a cross-sectional design, 103 described a single group prospective design, two described QEDs, and nine reports described an RCT, two of which were cost–benefit analyses. Three of the nine RCT reports (21–23) and both QEDs (24, 25) described distinct studies. Six additional reports were secondary analyses of the five distinct studies so were considered linked reports. Results are organized according to the five studies with linked reports in each section.

Descriptive statistics of the five studies included in this review are displayed in Table 1 and are organized by study design (i.e., RCT/QED). For the three RCTs, the average longest observation period was 18 months, 78% of the participants in these studies identified as non-White, 35% as female, and the average sample size was *N* = 221. For the two QEDs, the average longest observation period was 24 months, 78% of the participants in these studies identified as non-White, 95% as female, and the average sample size was *N* = 335. Details of the 11 reports generated from these five studies are summarized in Table 2, including group assignment, sources of participant recruitment, sample sizes, demographics, follow-up periods, retention rates, primary substances, and outcomes. Most studies concerned recovery houses following the Oxford House model (*k* = 4), while only one study examined a non-Oxford House model. Both models of recovery housing will be reviewed in turn.

**Table 1:** Quantity, nature, and brief description of published studies from a review of the evidence on recovery housing.

Type of study design	Number of studies k	Sample size mean N (range, SD)	Mean age (range, SD)	Mean % female (range, SD)	Mean % racial/ethnic minoritized (range, SD)	Longest follow-up months (mean, range, SD)	Primary substance of focus (n)					
							Alcohol	Mixed	Opioid	Stimulant	Cannabis	Other
Randomized controlled trial	3	221 (150–270, 63)	39 (37–40, 2)	35 (17–62, 24)	78 (68–89, 10.36)	18 (6–24, 10)	0	2	1	0	0	0
Quasi-experimental (non-randomized)	2	335 (200–470, 190)	40 (39–40, 0.18)	95 (90–100, 6.93)	74 (74–74, 0.21)	24 (24–24, 7)	0	2	0	0	0	0

**Table 2:** Eleven recovery housing reports generated by three randomized control trials and two quasi-experimental designs: description, demographics, retention, primary substance, and outcomes.

Article	Study design	Interventions	Sample					Follow-ups	Retention rate	Primary substance*	Outcomes
			Description	Size (N)	Gender	Race/ethnicity	Mean age (SD)				
Busto et al. (31) Parent Study: Jason et al. (22)	RCT	Exp #1: Oxford House Exp #2: Therapeutic Community Control: usual continuing care arrangements decided by participant	Post residential SUD treatment and criminal justice reentry/case management programs.	N=270	Male 83% Female 17%	African American 74.1% White 21.1% Hispanic 3.3% other 1.5%	39	6, 12, 18, 24 months	Exp #1: 82% Exp #2: 81% Control: 78%	Heroin 43.2% crack cocaine 28.9% alcohol 14.7% marijuana 7.1% polysubstance 5.6% amphetamine/ methamphetamine 0.4%	Exp #1: At baseline solitary activities (reading/writing) were the most frequently endorsed important activities (31%) which changed to education/work at follow-up (27.4%). Exp #2: At baseline solitary activities (reading/writing) were most frequently endorsed important activities (25%) which stayed the same at follow-up (14.1%) along with self-improvement (14.1%) and entertainment (14.5%). Control: At baseline solitary activities (reading/writing) were most frequently endorsed important activities (31.8%) which changed to education/work at follow-up (26.1%).
Chavarria et al. (27) Parent Study: Jason et al (21)	RCT	Exp: Oxford House Control: usual continuing care with case-worker referrals to treatment and community resources and arrangements decided by participants	Post residential treatment	N=150	Female 62% Male 38%	African American 77.3% White 11.3% Latino 8% Other 3.3%	37.07 (7.96)	6, 12, 18, 24 months	Exp: 89% Control: 86%	cocaine 60% alcohol 56% cannabis 38% heroin/opioids 35% sedatives 28% amphetamines 20%	Exp condition explained 63% of abstaining at 2 years.
Doleac et al. (30) reanalysis of parent study: Jason et al. (22)	RCT	Exp #1: Oxford House Exp #2: Therapeutic Community Control: usual continuing care arrangements decided by participant	Post residential SUD treatment and criminal justice reentry/case management programs.	N=270	Male 83% Female 17%	African American 74.1% White 21.1% Hispanic 3.3% other 1.5%	39	6, 12, 18, 24 months	Exp #1: 82% Exp #2: 81% Control: 78%	heroin 43.2% crack cocaine 28.9% alcohol 14.7% marijuana 7.1% polysubstance 5.6% amphetamine/ methamphetamine 0.4%	Oxford House: Significantly increased days incarcerated by 2.3 per month compared to control. Therapeutic Community: Significantly reduced days worked by 2.3 days per month and reduced income by $238 per month compared to control. No significant difference on days of alcohol use, days of drug use, illegal income, legal issues, or psychiatric hospitalizations. Outcomes not included in reanalysis: continuous alcohol use, continuous drug use, and cost-to-benefit.
Jason et al. (21) (Parent Study)	RCT	Exp: Oxford House Control: usual continuing care with case-worker referrals to treatment and community resources and arrangements decided by participants	Post residential substance use disorder treatment	N=150	Female 62% Male 38%	African American 77.3% White 11.3% Latino 8% other 3.3%	37.07 (7.96)	6, 12, 18, 24 months	over 90%	cocaine 60% alcohol 56% cannabis 38% heroin/opioids 35% sedatives 28% amphetamines 20%	Exp: 64.8% abstinent, monthly income $989.40, incarcerated 3%. All outcomes significant compared to usual continuing care. Control: 31.3% abstinent, monthly income $440.00, incarcerated 9%.
Jason et al. (26) Parent Study: Jason et al. (21)	RCT	Exp: Oxford House Control: usual continuing care with case-worker referrals to treatment and community resources and arrangements decided by participants	Post residential substance use disorder treatment	N=150	Female 62% Male 38%	African American 77.3% White 11.3% Latino 8% other 3.3%	37.07 (7.96)	6, 12, 18, 24 months	Exp: 89% Control: 86%	cocaine 60% alcohol 56% cannabis 38% heroin/opioid 35% sedative 28% amphetamine 20%	Exp: Any substance use past 6 months (31.3%), employed in past 30 days (76.1%), awaiting criminal charges in past 30 days (0%) self-regulation increased. All outcomes significant compared to control. Control: Any substance use past 6 months (64.8%), employed past 30 days (48.6%), awaiting criminal charges (5.6%). Additional outcomes not tested for significant difference at 24-months: 14 mothers assigned to Oxford House obtained custody of their children while one mother lost custody; in contrast to usual care, six mothers obtained custody of their children, and two mothers lost custody. Living in their own home was reported by 40% of Oxford House group compared to 13% of usual care.
Jason et al. (22) (Parent Study)	RCT	Exp #1: Oxford House Exp #2: Therapeutic Community Control: usual continuing care arrangements decided by participant	Post residential SUD treatment and criminal justice reentry/case management programs.	N=270	Male 83% Female 17%	African American 74.1% White 21.1% Hispanic 3.3% other 1.5%	39	6, 12, 18, 24 months	Exp #1: 82% Exp #2: 81% Control: 78%	heroin 43.2% crack cocaine 28.9% alcohol 14.7% marijuana 7.1% polysubstance 5.6% amphetamine/ methamphetamine 0.4%	Continuous abstinence from alcohol over two years significantly higher in Oxford House compared to Therapeutic Community and continuing care as usual: Exp #1: 66% Exp #2: 40% Control: 49% Money received from employment in the past 30 days significantly higher in Oxford House compared to Therapeutic Community: Exp #1: $680 Exp #2: $319 Control: $579 Number of paid workdays in the past 30 days significantly higher in Oxford House compared to Therapeutic community and continuing care as usual: Exp #1: 11.27 Exp #2: 6.37 Control: 8.45 Cost to benefit analysis showed net benefit per person in favor in Oxford House: Exp #1: $12,738 Exp #2: $ -7,510 Control: $3 No significant difference between groups on days using alcohol, days using other drugs, continuous abstinence from other drugs, illegal income obtained, legal issues, incarcerations, or psychiatric hospitalizations.
Jason et al. (24) (Parent Study)	QED	Exp: Oxford House Control: usual continuing care arrangements decided by participant	Women formerly incarcerated in the past two years	N=200	Female 100%	African American 74.5% other 25.5%	39.9 (8.58)	6, 12, 18, 24 months	Exp: 86% Control: 84%	heroin 47% crack/cocaine 29.5% alcohol 12.5% marijuana 7.5% other opiate 1.5% amphetamine 1% hallucinogen 1%	Death rates were higher in the control (4) compared to Oxford House (0) but not tested for significance. No difference between groups on substance use, employment, or arrests.
Lo Sasso et al. (28) Parent Study: Jason et al. (21)	Cost-Effectiveness of RCT	Exp: Oxford House Control: usual continuing care with case-worker referrals to treatment and community resources and arrangements decided by participants	Post residential treatment	N=129	Female 62% Male 38%	African American 77.3% White 11.3% Latino 8% other 3.3%	37.07 (7.96)	6, 12, 18, 24 months	over 90%	cocaine 60% alcohol 56% cannabis 38% heroin/opioid 35% sedative 28% amphetamine 20%	Net benefit of $29,022 per Oxford resident relative to usual care.
Majer et al. (25) (Parent Study)	QED	Exp #1: Oxford House Exp #2: Therapeutic Community Control: usual continuing care arrangements decided by participant	Women formerly incarcerated in the past two years (24) and post criminal justice system recruited from substance use disorder treatment facilities or reentry/case management programs (22)	N=470	Female 90.2% Male 9.8%	African American 74.2% White 21% other 4%	40.2 (9.1)	6, 12, 18, 24 months	QED: Exp: 86% Con: 84% RCT: Exp: 82% Con 1: 81% Con 2: 78%	heroin/opiates 45% cocaine 29.2% alcohol 13.6% cannabis 7.3% amphetamine/ methamphetamine.7%	Exp #1: Decreased number of days of substance use in the past 6 months from baseline (40.30) to 24 months (30.48), significant compared to Therapeutic Community and usual continuing care. Exp #2: Increased number of days of substance use in the past 6 months from baseline (35.20) to 24 months (47.17). Control: Increased number of days of substance use in the past 6 months from baseline (26.49) to 24 months (39.90).
Mueller et al, (29) Parent Study: Jason et al. (21)	RCT	Exp: Oxford House Control: usual continuing care with case-worker referrals to treatment and community resources and arrangements decided by participants	Post residential treatment	N=150	Female 62% Male 38%	African American 77.3% European American 11.3% Hispanic/Latino 8% Asian American 3.3%	37.1 (8.0)	6, 12, 18, 24 months	Exp: 89% Control: 86%	cocaine 60% alcohol 56% cannabis 38% heroin/opioid 35% sedative 28% amphetamine 20%	Exp: Number of people in recovery from alcohol in personal network significantly increased over time compared to control. Number of heavy drinkers in network stayed the same over time which was significantly different from control which increased. No significant difference between group on number of alcohol abstainers, number of light drinkers, number of moderate drinkers, network heterogeneity or size.
Tuten et al. (23) (Parent Study)	RCT	Exp #1: recovery housing Exp #2: recovery housing plus reinforcement-based treatment Control: usual continuing care with referrals to treatment and community resources and arrangements decided by participants	Patients who completed medication-assisted opioid detoxification	N=243	Male 74.1% Female 25.9%	African American 67.9%	38.7 (8.5)	1, 3, 6 months	Across 1,3,6 months 85% of all scheduled follow-up interviews and 77% of urine samples were collected	opioid dependence 100% cocaine dependence 66.1%	Exp #1: 37% drug abstinence significantly higher than control. Earned income significantly higher than control at 3 month but not 6 months. No significant difference in illegal activity compared to control. Exp #2: 50% drug abstinence significantly higher than control. Earned income significantly higher than control at 3 month and maintained at 6 months. No significant difference in illegal activity compared to control. Control: 13% drug abstinence.

Exp, experimental condition; Control, control condition; RCT, randomized control trial; QED, quasi-experimental design, *adds up to over 100% if participants endorsed all substances used as opposed to primary substance alone.

### 3.1 Oxford houses: randomized control trial #1

Oxford Houses are peer run, self-supporting homes that have no time limit for how long a resident can live there while abstinent from alcohol and other drugs. The first RCT studying Oxford Houses generated five publications that met inclusion criteria and are described here. Jason et al. (21) recruited participants from inpatient SUD treatment prior to discharge and assigned them to either Oxford Housing or continuing care as usual, which included no intervention above the pre-existing resources in the community (e.g., case-worker referrals with arrangements decided by the participant such as outpatient treatment, mutual-help groups, or alternative living arrangements). The sample mostly identified as African American (77%) and female (62%). At the two-year follow up results were statistically significant, Oxford House residents were twice as likely to be abstinent (65% vs. 31%), earned double the monthly income ($989 vs. $440), and had one-third of the incarceration rates (3% vs. 9%) compared to individuals assigned to continuing care. A secondary data analysis found additional support for similar measures. The Oxford House condition had half the rates of any substance use in the past 6 months (31% vs. 65%, *p* < 0.05), higher levels of employment (76% vs. 49%, *p* < 0.05), and fewer participants awaiting criminal charges (0% vs. 6%, *p* < 0.05) compared to continuing care as usual (26). Furthermore, the odds of a recurrence of substance use were reduced by 63% (*p* < 0.01) by Oxford House participation compared to continuing care (27). Many residents had left the home by the two-year follow-up (on good terms) which shows persistent and lasting effects of Oxford Houses on long-term recovery outcomes. The overall net benefit was higher for Oxford House (+$29,000 per resident, 95% CI: 12,292.19-45,751.81) relative to continuing care when accounting for the costs of healthcare, criminal activity, incarceration, alcohol or other drug use, and employment during this 2-year span (28) largely driven by differences in illegal activity. In addition, a secondary analysis showed that longer stays in an Oxford House were related to having more people in a social network who were in recovery, unlike continuing care, where the number of heavy drinkers in the network increased over time (29).

### 3.2 Oxford houses: randomized control trial #2

The second RCT examining Oxford House generated three publications. Jason et al. (22) randomized formerly incarcerated individuals recruited from inpatient SUD treatment facilities to three conditions: (1) Oxford House, (2) therapeutic community (a structured abstinence-based residential program with trained staff and site-manager supervision), or (3) continuing care as usual which included no intervention above the pre-existing resources in the community (arrangements decided by participants such as independent living or homeless shelters). The sample was predominantly African American (74%) males (83%) with an average of 10 previous incarcerations. After 2 years, participants assigned to the Oxford House group had the highest continuous alcohol abstinence rates (66%) compared to therapeutic communities (40%, *p* < 0.01) and continuing care (49%, *p* = 0.02). Additionally, compared to those randomized to a therapeutic community, Oxford House residents earned twice the monthly income ($680 vs. $319, *p* < 0.01) and worked nearly twice as many paid days in the past month (11 vs. 6, *p* < 0.01). Cost–benefit ratios also favored the Oxford House (+$12,738) over the therapeutic community (−$7,510, *p* < 0.01) and continuing care as usual (+$3,804, *p* < 0.01) models, mostly due to a reduction in costs associated with legal involvement. There was no difference between groups in the number of days of alcohol use, days of other drug use, legal issues, incarcerations, psychiatric hospitalizations, or illegal income. Although continuous alcohol abstinence rates were highest in the Oxford House condition, alcohol as a primary substance was reported by only 15% of the participants. There was no difference between groups in continuous abstinence from other drugs, which was the primary substance for 85% of the study sample (43% heroin, 29% crack cocaine, 7% marijuana, and 6% polysubstance, <1% amphetamine/methamphetamine).

In a reanalysis and extension of Jason et al. (22) and Doleac et al. (30) arrived at different conclusions regarding days incarcerated after implementing a series of substantially altered conceptual and methodological steps compared to the original publication. The authors reported that participants in Oxford Housing had significantly increased days incarcerated by 2 per month compared to continuing care as usual (*p* < 0.10), unlike the original study which found no difference. Several outcomes were not included in the reanalysis such as the cost-to-benefit ratio, or the timeline follow-back measures of continuous alcohol use or other drug use.

A secondary data analysis of this RCT (31) qualitatively examined activities considered to be important among residents. At baseline, solitary activities such as reading and writing were the top activities. After 2 years, priorities significantly changed to education and work except in the therapeutic community condition which prioritized solitary activities, self-improvement and entertainment.

In summary of RCT #2, the Oxford House showed positive effects on a marginalized, formerly incarcerated population with regards to continuous alcohol abstinence, monthly income, paid workdays, and cost to benefit ratio. Days incarcerated had mixed findings, however, other indicators of criminal legal involvement, such as length of time of the most recent incarceration and illegal income, directionally favored Oxford House over time (32) and thus did not support the validity of the overall observation of criminal legal involvement as found in the reanalysis and extension. Importantly, drugs other than alcohol were the primary substance reported by 85% of the sample and there was no difference between groups in continuous abstinence from other drugs after 2 years. Participants entering the Oxford House condition were leaving controlled environments (i.e., residential treatment and re-entry programs) which may explain the lack of change from baseline to follow-up if their levels of substance use were already low upon entering the house. The high number of previous incarcerations means this group has many barriers to the recovery process and the Oxford house was able to improve continuous alcohol abstinence, income, and days worked beyond that of therapeutic communities or continuing care as usual following inpatient treatment for SUD.

### 3.3 Oxford houses: quasi-experimental #1

In a quasi-experimental study, Jason et al. (24) assigned formerly incarcerated women with a history of SUD to Oxford Housing versus continuing care as usual. The assignment was non-random as it was based on Oxford Housing availability at the time of participant recruitment. Continuing care as usual involved offering no intervention above what was currently in the community after completing treatment or leaving incarceration (e.g., living with a relative, outpatient treatment). Similar to the results of formerly incarcerated African American men, formerly incarcerated African American women reported high proportions of primary substances being other drugs (47% heroin, 30% crack cocaine, 8% marijuana, 2% other opiates) rather than alcohol (13%). After 2 years, the Oxford House condition had zero deaths compared to four deaths in continuing care as usual. Although the difference in mortality was not tested for statistical significance the Oxford Houses appear to be a lifesaving recovery support service for formerly incarcerated women. There were no differences in substance use, employment, or arrests when assigned to Oxford Housing versus continuing care as usual, among women released from detention.

### 3.4 Oxford houses: quasi-experimental #2

Majer et al. (25) combined samples from an RCT of Oxford House, therapeutic communities, and continuing care as usual/no intervention (22) and the QED of Oxford House and continuing care as usual/no intervention described above (24) to create an analytic sample of mostly African American (74%) women (90%) who were formerly incarcerated and leaving treatment. Forty-five percent reported heroin/opiates as their primary substance, 29% reported crack/cocaine, and 14% reported alcohol. At the two-year follow-up, Oxford House participants had decreased the number of days of substance use in the past 6 months by 10 days, compared to those in the therapeutic community which increased by 12 days, and continuing care which increased by 13 days (*p* < 0.03).

### 3.5 Recovery housing (non-oxford): randomized control trial #1

Tuten et al. (23) randomized patients who completed medically-managed withdrawal for opioid use disorder to, (1) a recovery housing condition where rent payment was contingent on twice-weekly negative opioid and cocaine urine samples, (2) a recovery housing plus reinforcement-based therapy condition, or (3) continuing care as usual which consisted of referrals to treatment and community resources with arrangements decided by the participant. Participants were mostly African American (68%), male (74%), and 65% had a cocaine use disorder. After 6 months, significantly higher drug abstinence rates were observed among participants randomized to recovery housing plus reinforcement-based therapy (50%), and among those in the recovery housing condition alone (37%), relative to continuing care as usual (13%, *p* < 0.001). Income was highest in both recovery housing conditions compared to continuing care as usual at 3 months and then recovery housing plus treatment at 6 months. There was no difference in illegal activity among conditions. This study identified two pathways to recovery for opioid use disorder.

## 4 Discussion

### 4.1 Implications of existing recovery housing research

With five original studies (only one of which is a non-oxford house model) and eleven total reports, the quantity of the evidence base is low. As such it is too soon to draw confident conclusions about this class of service across the board. At the same time, the quality of the available evidence is moderately strong given findings are drawn from longitudinal RCTs and QEDs, including those with active comparisons, and follow-up durations that lasted for years. Findings on the efficacy of recovery housing are strong for cost effectiveness and moderately strong for 3 outcome domains: (1) Substance use, and more consistently for studies that had more male participants. This is tempered by the fact that one Oxford House trial comprised of mostly men (85%) had no effect on other drug use which was the primary substance of most of the participants. In contrast, the non-Oxford House model had considerable opioid and cocaine abstinence rates and consisted of mostly men. (2) Income, especially for studies that included men for both Oxford and non-Oxford House models. (3) Employment, which was consistent in Oxford House studies that included men. Oxford House studies of almost all women (90% or more) who were formerly incarcerated showed no benefit for employment and income relative to continuing care as usual/no intervention or therapeutic communities. In addition, overall criminal legal involvement emerged as weak to moderate due to mixed findings on incarceration across recovery housing studies (i.e., reduced rates, no difference relative to comparison, and increased rates were found), yet reduced rates of awaiting criminal charges. Overall, the studies that included men favored recovery housing, but those tested on formerly incarcerated study samples who had high proportions of women (90% or more) showed positive but minimal benefit and findings were inconsistent. Therefore, the potential lifesaving benefit among formerly incarcerated women was notable. The global rating for empirical support that summarizes quantity, quality, and support for the recovery housing model is moderate.

A glaring finding from the description of published studies is that about 75% of the recovery housing participants were identified as African American. This high proportion is not explained by their representativeness in the U.S. population (14%) or their likelihood of having a SUD (33) but can be explained by their likelihood of being incarcerated for drug use compared to their White counterparts. The “war on drugs” populated prisons and jails with people of color. The U.S. is moving towards a public health approach as part of the national drug control strategy however the effects still linger. Today, 5% of people who use drugs identify as Black, yet Black Americans represent 33% of those incarcerated for drug offences (34). Most studies recruited participants from post incarceration programs which explains the overrepresentation of Black participants. These participants may have survived the war on drugs and now recovery housing is acting as a protective structural mechanism of health and health equity. Recovery housing has been shown to accelerate the recovery process faster for Black residents compared to other racial/ethnic groups (35). Regarding retention, a study of Oxford Houses found that Black residents tended to stay longer than others (36), yet a study of recovery houses found they were less likely to be retained at the same rate compared to their non-Black counterparts (37). This may reflect the differential impact of peer run (i.e., Oxford House) versus monitored and supervised homes on Black residents’ retention. At a time when Black Americans between the ages of 55–64 have the highest rates of fatal overdose, despite declines among their White counterparts (38), there is an urgent need to understand how post-incarcerated individuals and African Americans can be linked to and derive an increased benefit from recovery housing models, particularly among women.

### 4.2 Future directions

A number of suggestions for future research can advance the science of recovery housing. Every study in this review, with the exception of one RCT, examined the Oxford House model, which is a peer run house. Recovery housing has been classified according to types of support that extend beyond peer run including monitored, supervised, and clinical as referenced in updated patient placement guidelines (16). Comparative effectiveness has been used to show the measurable benefit of adding recovery housing to treatment (39). Still, many questions remain about the utility of all levels of support, for whom they are most beneficial depending on clinical (e.g., primary substance, level of severity) or demographic factors (e.g., gender), as well as where on the care continua (e.g., post medically managed withdrawal), when in the recovery process (e.g., initial, early), and for what duration. Cost-effectiveness research on levels of support will yield useful evidence to inform both housing and treatment providers as well as supply third party payers with the information required to offer recovery housing as a health insurance benefit.

One study of the Oxford House model showed positive effects on alcohol abstinence but no effect on other drug abstinence. In contrast, a non-Oxford House model of recovery housing for participants with opioid use disorder had moderately strong effects on drug abstinence. The differential effectiveness that recovery housing models can have on primary substance will need to be explored so matches between residents and houses can be made with precision.

Evidence suggests that recovery housing may not consistently benefit formerly incarcerated women the same as men. Yet, one Oxford House study of women showed potentially lifesaving benefits with zero deaths after 2 years compared to four in continuing care as usual. Additionally, 14 mothers in Oxford House obtained custody of their children compared to six (directional effect not tested for statistical significance). Recovery housing models can be adapted to better serve the needs of women with carceral exposure. Studies have shown that men and women face different life contexts and challenges and utilize and benefit from treatments in different ways (40). Evidence suggests that when you add motivational interviewing case management to recovery housing, legally involved women have higher abstinence 1 year later (52). Managers of women’s houses can be trained in motivational techniques including group engagement to facilitate retention (41). Additionally, case-managed approaches can be offered within the range of services in recovery housing to better service the needs of women. Issues of bias, stigma and mistrust have been more pervasive in the recovery narratives of women and non-White women thereby offering an actionable target to reduce barriers and improve recovery outcomes (42, 43).

Although an effective duration of recovery housing has not been rigorously studied, correlational evidence suggests between three to 6 months (26, 44–47). However, there are policies that restrict the duration of residency for reasons not informed by an effective dosage. Additionally, if health insurance is to offer recovery housing as a benefit, evidence on an effective duration will need to inform decisions. Pilot studies that randomize participants to durations of residency (e.g., 3,6, 12 months) will serve to inform unanswered questions regarding an effective dosage. Additionally, policy changes that allow restrictions on duration of residency to be manipulated (i.e., as levels of an independent variable) and then outcomes evaluated, will serve to inform evidenced based policy approaches that sustain long-term recovery.

No studies have been done to compare recovery housing alongside front line pharmacotherapies for substance use disorders. Medication trials are being conducted separate from recovery housing which fails to provide comparative effect sizes. This approach will allow providers and residents to make evidenced based decisions about the likelihood of remission for each pathway to recovery and provide more options for patient centered approaches.

Additional resident outcomes also warrant examination in RCTs (e.g., recovery capital, quality of life). Recovery capital is an emerging international construct and organizing framework that describes the internal and external resources brought to bear on a recovery attempt. Evidence suggests that when residents with low recovery capital were targeted for an intensive intervention consisting of support and assertive linkage it increased engagement, retention, and changes in recovery capital relative to a comparison group (48).

No rigorous studies have examined recovery housing and Housing First. Housing First targets chronically unhoused people, with or without SUD, with the goal of achieving rapid housing using a low threshold approach. In contrast, recovery housing targets people with SUD using a structured abstinent based living environment with the goal of remission and stable long-term recovery. Largely these interventions are designed to achieve different endpoints. RCTs of Housing First have shown no reduction in symptomology associated with substance use or mental health (49, 50). However, the federal government is increasingly interested in this intersection and creating housing continuums that offer both services with warm handoffs and linkages between them (10, 14). Science has offered little evidence to guide this strategy. Future studies should seek to inform the coordination of these services at the level of practice, policy, and funding.

### 4.3 Bias and limitations

The results of this systematic review should be considered in the context of the following limitations. Only studies published in English were included in this review which could lead to the potential for publication or reporting bias as some regionally specific studies are not represented in the findings. This limits generalizability of the findings to non-English speaking areas. Also, only a single author screened the studies for inclusion and extracted the study data. Future systematic reviews of the scientific literature on recovery housing should include a second reviewer to support validation. Only RCTs and QEDs were included in this review – though this ensured evaluation of the highest quality studies and allowed for more rigorous interpretation of results, it limited the number of studies from which conclusions were drawn. With the limited overall total sample size of *N* = 1,333 individuals across just 5 studies, as well as variability in the sample characteristics, follow-up durations, and types of housing models tested, it is difficult to conclude with high confidence much about the general public health and recovery supportive utility of the recovery housing model. That said, when the evidence base is viewed as a whole and considered from multiple theoretical and evidentiary viewpoints -single group prospective, retrospective, cross-sectional, as well as the comparative effectiveness, efficacy, and cost-effectiveness studies reviewed in this paper- there is strong consistency, coherence, and convergence, across findings, suggesting recovery housing is at least a promising intervention that can help many people suffering from SUD who have low resources and high levels of clinical pathology and prior criminal legal involvement to increase their chances of remission and stable recovery.

## 5 Conclusion

Similar to other chronic health conditions, continuing care models are a critical component in recovery from SUD with recovery housing being the most widely available form of recovery support infrastructure (12). The literature on comparative efficacy suggests that recovery housing can lead to improvements in substance use, income, and employment. Criminal legal involvement showed mixed results, but overall trends suggest recovery housing to be equivalent to comparison conditions or more beneficial. The global rating for empirical support based on quantity, quality, and support for recovery housing is moderate. Future research should extend beyond Oxford House models, focus on women with carceral exposure, study the effect of recovery housing on primary substance, understand who needs what level of recovery housing support, test recovery housing alongside front-line pharmacotherapies, study optimal durations of recovery housing, study the intersection of recovery housing and Housing First, and include cost-effectiveness. Additionally, future reviews of the scientific literature on recovery housing should include mechanisms of behavior change, and studies that test recovery housing as an add-on or test adding on an intervention to recovery housing.

## Data Availability

The original contributions presented in the study are included in the article/Supplementary material, further inquiries can be directed to the corresponding authors.
