# Supplementary material for: Recovery housing for substance use disorder: a systematic review
Source: Front Public Health. 2025 Mar 6;13:1506412. doi: 10.3389/fpubh.2025.1506412 (PMC11922849; doi:10.3389/fpubh.2025.1506412)
Supplement: Supplementary file 1 [file Data_Sheet_1.DOCX]

**Appendix A.**

**Pubmed**

((“communal living”[Title/Abstract] OR “dry home*”[Title/Abstract] OR “dry hous*”[Title/Abstract] OR “halfway home*”[Title/Abstract] OR “halfway hous*”[Title/Abstract] OR “halfway residence*”[Title/Abstract] OR “oxford home*”[Title/Abstract] OR “oxford hous*”[Title/Abstract] OR “recovery home*”[Title/Abstract] OR “recovery hous*”[Title/Abstract] OR “recovery residence*”[Title/Abstract] OR “sober home*”[Title/Abstract] OR “sober hous*”[Title/Abstract] OR “sober living”[Title/Abstract] OR “transitional home*”[Title/Abstract] OR “transitional hous*”[Title/Abstract] OR “wet home*”[Title/Abstract] OR “wet hous*”[Title/Abstract] OR domiciliary[Title/Abstract])) AND ((recovery[Title/Abstract] OR remission[Title/Abstract] OR abstinence[Title/Abstract] OR "harm reduction"[Title/Abstract] OR “substance abuse”[Title/Abstract] OR “substance misuse”[Title/Abstract] OR “substance dependence”[Title/Abstract] OR “drug dependence”[Title/Abstract] OR “substance use disorder”[Title/Abstract] OR “alcohol use disorder”[Title/Abstract] OR “drug use disorder”[Title/Abstract] OR alcohol*[Title/Abstract] OR marijuana[Title/Abstract] OR “THC”[Title/Abstract] OR cannabis[Title/Abstract] OR cocaine[Title/Abstract] OR heroin[Title/Abstract] OR opioid*[Title/Abstract] OR opiate*[Title/Abstract] OR narcotic*[Title/Abstract] OR amphetamine*[Title/Abstract] OR methamphetamine*[Title/Abstract] OR benzodiazepine*[Title/Abstract] OR barbiturate*[Title/Abstract] OR hallucinogen*[Title/Abstract] OR inhalant*[Title/Abstract] OR steroid*[Title/Abstract] OR “club drug*”[Title/Abstract] OR ecstasy[Title/Abstract] OR “MDMA”[Title/Abstract] OR stimulant*[Title/Abstract] OR cost-benefit[Title/Abstract] OR cost-offset[Title/Abstract] OR cost-effectiveness[Title/Abstract]) OR “cost benefit”[Title/Abstract] OR “cost offset”[Title/Abstract] OR “cost effectiveness”[Title/Abstract]))

**Embase**

(‘communal living’:ab,ti OR ‘dry home*’:ab,ti OR ‘dry hous*’:ab,ti OR ‘halfway home*’:ab,ti OR ‘halfway hous*’:ab,ti OR ‘halfway residence*’:ab,ti OR ‘oxford home*’:ab,ti OR ‘oxford hous*’:ab,ti OR ‘recovery home*’:ab,ti OR ‘recovery hous*’:ab,ti OR ‘recovery residence*’:ab,ti OR ‘sober home*’:ab,ti OR ‘sober hous*’:ab,ti OR ‘sober living’:ab,ti OR ‘transitional home*’:ab,ti OR ‘transitional hous*’:ab,ti OR ‘wet home*’:ab,ti OR ‘wet hous*’:ab,ti OR domiciliary:ab,ti) AND (recovery:ab,ti OR remission:ab,ti OR abstinence:ab,ti OR 'harm reduction':ab,ti OR ‘substance abuse’:ab,ti OR ‘substance misuse’:ab,ti OR ‘substance dependence’:ab,ti OR ‘drug dependence’:ab,ti OR ‘substance use disorder’:ab,ti OR ‘alcohol use disorder’:ab,ti OR ‘drug use disorder’:ab,ti OR alcohol*:ab,ti OR marijuana:ab,ti OR ‘THC’:ab,ti OR cannabis:ab,ti OR cocaine:ab,ti OR heroin:ab,ti OR opioid*:ab,ti OR opiate*:ab,ti OR narcotic*:ab,ti OR amphetamine*:ab,ti OR methamphetamine*:ab,ti OR benzodiazepine*:ab,ti OR barbiturate*:ab,ti OR hallucinogen*:ab,ti OR inhalant*:ab,ti OR steroid*:ab,ti OR ‘club drug*’:ab,ti OR ecstasy:ab,ti OR ‘MDMA’:ab,ti OR stimulant*:ab,ti OR cost-benefit:ab,ti OR cost-offset:ab,ti OR cost-effectiveness:ab,ti OR ‘cost benefit’:ab,ti OR ‘cost offset’:ab,ti OR ‘cost effectiveness’:ab,ti)

**CINAHL**

AB (“communal living” OR “dry home*” OR “dry hous*” OR “halfway home*” OR “halfway hous*” OR “halfway residence*” OR “oxford home*” OR “oxford hous*” OR “recovery home*” OR “recovery hous*” OR “recovery residence*” OR “sober home*” OR “sober hous*” OR “sober living” OR “transitional home*” OR “transitional hous*” OR “wet home*” OR “wet hous*” OR domiciliary) AND AB ( recovery OR remission OR abstinence OR "harm reduction" OR “substance abuse” OR “substance misuse” OR “substance dependence” OR “drug dependence” OR “substance use disorder” OR “alcohol use disorder” OR “drug use disorder” OR alcohol* OR marijuana OR “THC” OR cannabis OR cocaine OR heroin OR opioid* OR opiate* OR narcotic* OR amphetamine* OR methamphetamine* OR benzodiazepine* OR barbiturate* OR hallucinogen* OR inhalant* OR steroid* OR “club drug*” OR ecstasy OR “MDMA” OR stimulant* OR cost-benefit OR cost-offset OR cost-effectiveness OR “cost benefit” OR “cost offset” OR “cost effectiveness” )

AB (“communal living” OR “dry home*” OR “dry hous*” OR “halfway home*” OR “halfway hous*” OR “halfway residence*” OR “oxford home*” OR “oxford hous*” OR “recovery home*” OR “recovery hous*” OR “recovery residence*” OR “sober home*” OR “sober hous*” OR “sober living” OR “transitional home*” OR “transitional hous*” OR “wet home*” OR “wet hous*” OR domiciliary) AND TI ( recovery OR remission OR abstinence OR "harm reduction" OR “substance abuse” OR “substance misuse” OR “substance dependence” OR “drug dependence” OR “substance use disorder” OR “alcohol use disorder” OR “drug use disorder” OR alcohol* OR marijuana OR “THC” OR cannabis OR cocaine OR heroin OR opioid* OR opiate* OR narcotic* OR amphetamine* OR methamphetamine* OR benzodiazepine* OR barbiturate* OR hallucinogen* OR inhalant* OR steroid* OR “club drug*” OR ecstasy OR “MDMA” OR stimulant* OR cost-benefit OR cost-offset OR cost-effectiveness OR “cost benefit” OR “cost offset” OR “cost effectiveness” )

TI (“communal living” OR “dry home*” OR “dry hous*” OR “halfway home*” OR “halfway hous*” OR “halfway residence*” OR “oxford home*” OR “oxford hous*” OR “recovery home*” OR “recovery hous*” OR “recovery residence*” OR “sober home*” OR “sober hous*” OR “sober living” OR “transitional home*” OR “transitional hous*” OR “wet home*” OR “wet hous*” OR domiciliary) AND AB ( recovery OR remission OR abstinence OR "harm reduction" OR “substance abuse” OR “substance misuse” OR “substance dependence” OR “drug dependence” OR “substance use disorder” OR “alcohol use disorder” OR “drug use disorder” OR alcohol* OR marijuana OR “THC” OR cannabis OR cocaine OR heroin OR opioid* OR opiate* OR narcotic* OR amphetamine* OR methamphetamine* OR benzodiazepine* OR barbiturate* OR hallucinogen* OR inhalant* OR steroid* OR “club drug*” OR ecstasy OR “MDMA” OR stimulant* OR cost-benefit OR cost-offset OR cost-effectiveness OR “cost benefit” OR “cost offset” OR “cost effectiveness”)

TI (“communal living” OR “dry home*” OR “dry hous*” OR “halfway home*” OR “halfway hous*” OR “halfway residence*” OR “oxford home*” OR “oxford hous*” OR “recovery home*” OR “recovery hous*” OR “recovery residence*” OR “sober home*” OR “sober hous*” OR “sober living” OR “transitional home*” OR “transitional hous*” OR “wet home*” OR “wet hous*” OR domiciliary) AND TI ( recovery OR remission OR abstinence OR "harm reduction" OR “substance abuse” OR “substance misuse” OR “substance dependence” OR “drug dependence” OR “substance use disorder” OR “alcohol use disorder” OR “drug use disorder” OR alcohol* OR marijuana OR “THC” OR cannabis OR cocaine OR heroin OR opioid* OR opiate* OR narcotic* OR amphetamine* OR methamphetamine* OR benzodiazepine* OR barbiturate* OR hallucinogen* OR inhalant* OR steroid* OR “club drug*” OR ecstasy OR “MDMA” OR stimulant* OR cost-benefit OR cost-offset OR cost-effectiveness OR “cost benefit” OR “cost offset” OR “cost effectiveness”)

**CENTRAL (Cochrane Registry)**

Same as for CINAHL

**PsycINFO**

Same as for CINAHL
